# Appendicitis within Morgagni Hernia and simultaneous Paraesophageal Hernia

**DOI:** 10.1186/1471-2482-15-15

**Published:** 2015-02-02

**Authors:** Anna Bettini, Jesus G Ulloa, Hobart Harris

**Affiliations:** UCSF Department of Surgery, University of California, 513 Parnassus Avenus, S–321, San Francisco, CA 94143-0001 USA

**Keywords:** Morgagni, Paraesophageal, Defect and appendicitis

## Abstract

**Background:**

Morgagni hernia is a congenital diaphragmatic defect that rarely presents with symptomatic findings in adults. The presence of one diaphragmatic defect may decrease the occurrence of a separate diaphragmatic defect. Appendicitis may be a unique presentation of incarcerated bowel in a Morgagni defect.

**Case presentation:**

Review of recent literature and presentation of a patient with Morgagni defect. Only five cases of simultaneous Morgagni hernia and paraesophageal hernia have been described in the English-language literature since 1958. Here, we report the first case of acute appendicitis within an incarcerated right Morgagni hernia in a 76-year-old patient who also had a paraesophageal hernia.

**Conclusion:**

This case illustrates that there is no role for watchful waiting in the management of Morgagni Defects when diagnosed in adult patients.

## Background

The coexistence of two non-traumatic diaphragmatic hernias is rare; only five cases of combined Morgagni and paraesophageal hernias have been described in the English-language literature [1–5]. Focal weakness in the diaphragmatic musculature and a concomitant increase in intra-abdominal pressure contribute to the formation of diaphragmatic hernias [3, 4]. The presence of a large diaphragmatic hernia, by reducing intra-abdominal pressure, would therefore decrease the likelihood of a second diaphragmatic hernia [3, 4].

Paraesophageal hiatal hernias account for approximately 14% of hiatal hernias and occur most commonly in adults [3]. The defect is situated anterior to esophagus and may cause characteristic symptoms of chest pain, dysphagia, regurgitation, and occult bleeding [2]. Herniation of abdominal contents into the thorax via a subcostosternal defect, as first described by Morgagni in 1769, is a congenital problem that rarely presents in adults and accounts for 3–5% of diaphragmatic hernias [3, 6].

In all five cases of simultaneous Morgagni hernia and paraesophageal hernia, the paraesophageal defect led to the main symptomatic complaint. Surgical intervention focused on repair of the dual diaphragmatic defects, with one case citing the incidental finding of Morgagni defect at time of paraesophageal repair. In this case report, we describe the surgical procedure and outcome of a patient with acute appendicitis within an incarcerated right Morgagni hernia combined with a paraesophageal hernia.

## Case report

The patient was a 76-year-old man with abdominal pain who was transferred to our hospital in August, 2013 from another institution. He had experienced 48 hours of obstipation, and computed tomography of the abdomen was concerning for an incarcerated Morgagni’s hernia. He had a 10-month history of intermittent abdominal discomfort after eating large meals. Evaluation at another institution in 2012 had revealed a right Morgagni’s hernia that was managed non-operatively. He also had a longstanding history of gastroesophageal reflux disease and a known moderate-sized paraesophageal hernia.

**Figure 1:**
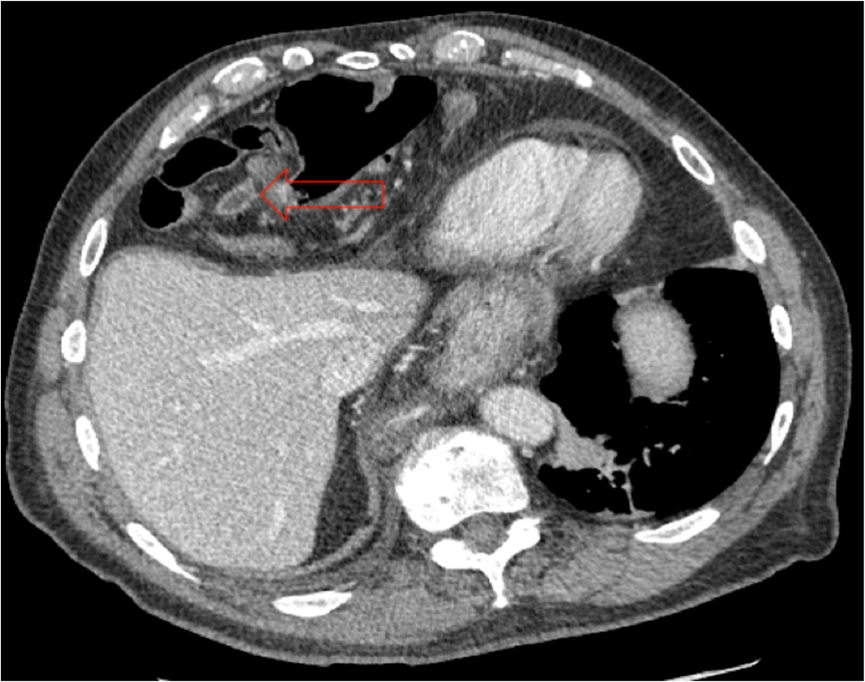
**Computed tomography scan obtained at our institution 8/2013, with arrow showing appendiceal inflammation within Morgagni defect.** Computed tomography scan obtained at our institution 8/2013, with arrow showing appendiceal inflammation within Morgagni defect.

**Figure 2:**
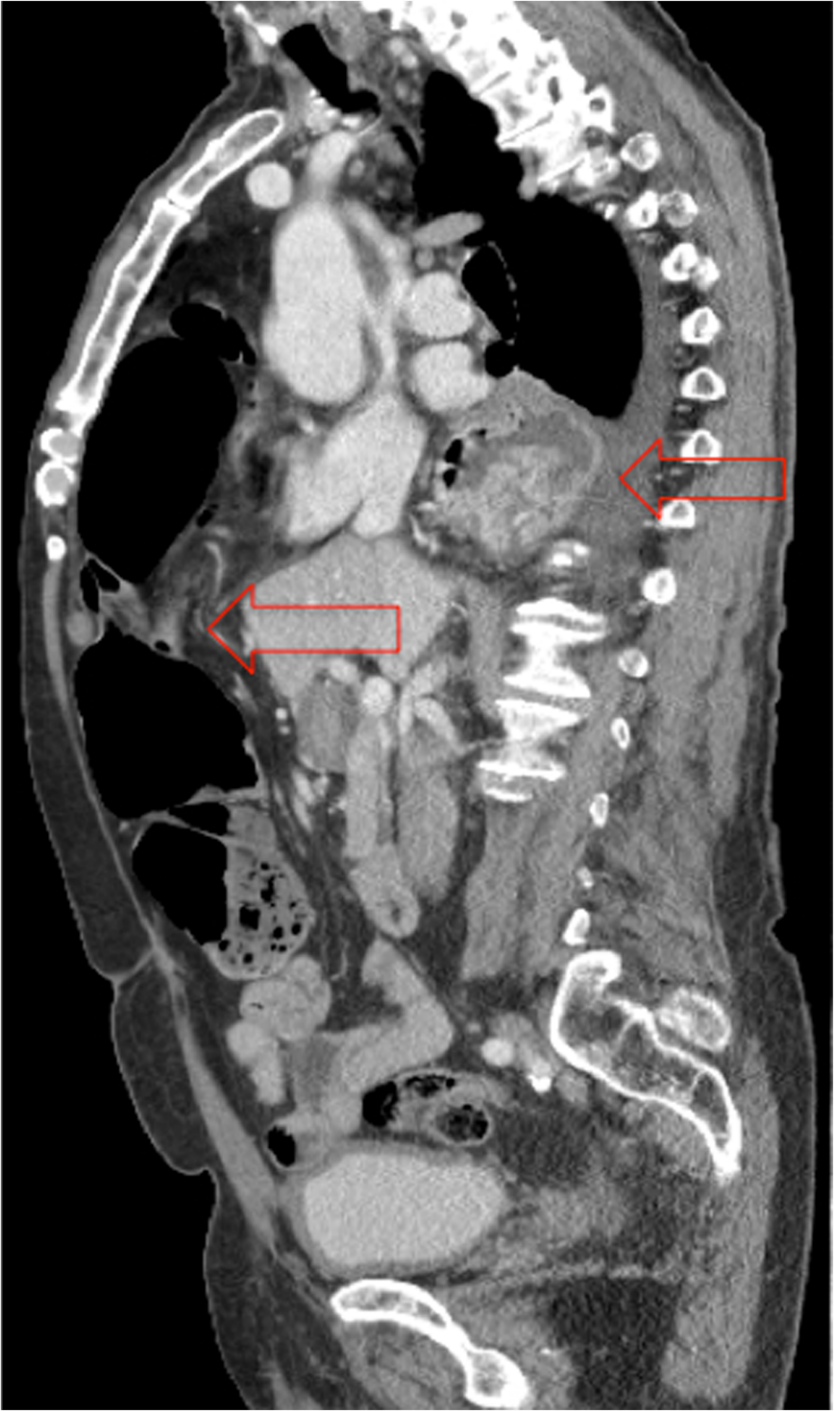
**Computed tomography scan obtained 8/2013, sagittal view with arrows showing anterior Morgagni defect and posterior paraesophageal hernia.** Computed tomography scan obtained 8/2013, sagittal view with arrows showing anterior Morgagni defect and posterior paraesophageal hernia.

A physical examination revealed decreased breath sounds in the base of the right lung and diffuse abdominal tenderness with guarding. There was focal tenderness at the right lateral chest wall. Computed tomography (CT) scans of the chest, abdomen and pelvis obtained for operative planning showed a large right Morgagni hernia containing small bowel, large bowel, and appendix with interval development of acute appendicitis when compared to CT scans obtained at the referring facility (Figures 1 and 2). A moderate paraesophageal hernia was stable in size compared to its appearance in prior studies. These physical and imaging findings indicated the need for surgical intervention.

**Figure 3:**
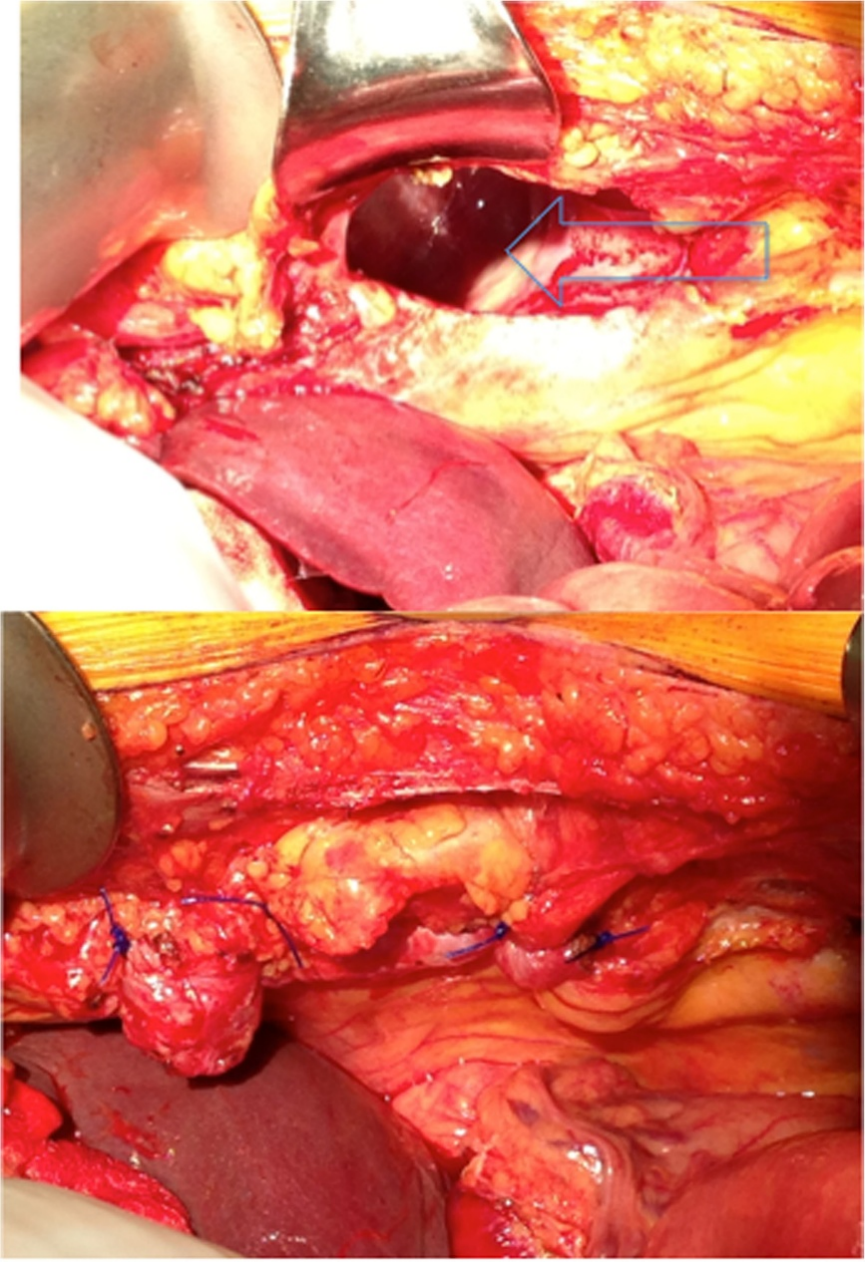
**Intraoperative Morgagni defect before (above) and after (below) primary repair.** Defect opening anterior to liver with central retractor overlying xiphoid process. Intraoperative Morgagni defect before (above) and after (below) primary repair.

We chose an upper midline laparotomy over a laparoscopic approach, given the possibility of ischemic or necrotic bowel. There was a 4 cm by 6 cm anterior defect 5 cm right of midline, anterior to the liver (Figure 3), containing approximately 100 cm of distal ileum, cecum, appendix, and a portion of the ascending and transverse colon. The cecum was dilated but viable and the appendix had a dilated tip without evidence of perforation.

The Morgagni hernia contents were reduced and an appendectomy was performed. The paraesophageal hernia was reduced, the hiatal defect was repaired and a Nissen fundoplication was performed. The Morgagni defect was repaired primarily with interrupted prolene sutures without removal of the hernia sac (Figure 3).

Postoperatively, the patient’s course was complicated by a pulmonary embolism that required anticoagulation with a direct thrombin inhibitor because of heparin resistance, new onset atrial fibrillation secondary to volume overload that improved with aggressive diuresis, *Clostridium difficile* colitis treated with oral vancomycin, and ileus that resolved with nasogastric decompression and bowel rest. He was discharged home on postoperative day 14. The final pathology report confirmed acute appendicitis and periappendiceal inflammation. At his follow-up visit, the patient was doing well and eating a normal diet without dysphagia or persistent gastroesophageal reflux.

## Discussion

This case is striking because of the combination of two diaphragmatic defects, together with an appendiceal pathology. We are not aware of another such case in the English-language literature. Our patient’s chronic, intermittent symptoms, and ultimate trajectory towards incarcerated bowel and acute appendicitis within the hernia, supports the existing teaching that there is no role for watchful waiting in the case of Morgagni hernia. Surgical repair of Morgagni hernia is indicated both in asymptomatic and symptomatic patients to avoid morbidity due to incarceration or ischemic changes of abdominal contents in the chest [6–8].

Almost any non-retroperitoneal abdominal organ can be found within a Morgagni hernia, but the contents most commonly found during surgical exploration are omentum exclusively (31%), omentum and colon (29%), stomach (15%) and small bowel (11%) [7]. The defect is congenital, though trauma or factors that raise intra-abdominal pressure such as obesity, pregnancy, chronic constipation, and chronic cough, are implicated in hernia formation [6, 7]. As our case shows, however, simultaneous large defects are possible in the absence of predisposing factors, such as obesity or trauma.

Most Morgagni hernias come to clinical attention in childhood. It is a common belief that adult patients often are typically asymptomatic at the time of presentation, with the defect discovered incidentally on imaging during workup for other problems [4, 9]. However*,* a review of 298 cases of adult Morgagni hernia revealed only 28% patients were asymptomatic at the time of presentation [7]. Most frequently, patients presented with respiratory complaints, including persistent cough, dyspnea, or exercise intolerance. Abdominal discomfort and focal pain in the chest wall are also possible, as in our patient.

Once a Morgagni hernia is diagnosed, surgery is indicated because of the risk of incarcerated omentum, small bowel, colon or other complications such as intra-abdominal pathology mistaken for primarily thoracic pathology [6–8]. In a review of 298 cases of adult Morgagni hernias, Horton et al. found 72% of patients presented with pain and pulmonary complaints directly related to their Morgagni hernia [7]. Minneci et al. reported a case series of twelve patients where the most common contents in the Morgagni hernia sac were omentum (94%), colon (58%), stomach (25%) and small bowel (17%) and conclude prompt surgical repair is paramount to avoiding future patient morbidity, as the risk of incarceration is not trivial [8]. There is currently no consensus on the best surgical approach, partly because the condition is rare. The traditional approach for Morgagni hernia repair has been a midline laparotomy, but laparoscopic access may eventually replace it as practitioners gain more experience [8]. In our patient, based on surgeon preference for operative exposure, an upper midline laparotomy provided good common access to bilateral hernias in preparation for possible intraoperative findings of ischemic or necrotic bowel.

Since the 1990s, thoracoscopic and laparoscopic methods of Morgagni hernia repair have been described as having shorter hospital stays and fewer complications than with laparotomy or thoracotomy, as well as successful long-term outcomes [10]. However, the higher complication rate and longer hospital stay after laparotomy may be explained by the fact that most emergency operations are performed with this approach, whereas minimally invasive repairs are primarily used in non-emergent settings [7, 11].

## Conclusions

In conclusion, the combination of two diaphragmatic hernias and appendicitis make this case unusual, and provide further support for the existing teaching that there is no a role for watchful waiting in the case of Morgagni hernia. Instead, asymptomatic and symptomatic patients require surgery to avoid morbidity as a result of incarceration or compromise of abdominal contents in the chest.

## Consent

Written informed consent was obtained from the patient for publication of this Case report and any accompanying images. A copy of the written consent is available for review by the Editor of this journal. No ethics committee approval required for clinical case report.
